# Predictive factors over time of health-related quality of life in COPD patients

**DOI:** 10.1186/s12931-020-01395-z

**Published:** 2020-06-05

**Authors:** Cristóbal Esteban, Inmaculada Arostegui, Amaia Aramburu, Javier Moraza, Josu Najera-Zuloaga, Myriam Aburto, Susana Aizpiri, Leyre Chasco, José M. Quintana

**Affiliations:** 1 Respiratory Department, Hospital Galdakao-Usansolo, Galdakao, Bizkaia Spain; 2 Department of Applied Mathematics, Statistics and Operative Research, University of the Basque Country (UPV/EHU) and Basque Center for Applied Mathematics (BCAM), Bizkaia, Spain; 3 Department of Mechanics, Design and Industrial Organization, Universidad de Deusto, Bizkaia, Spain; 4 Research Unit, Hospital Galdakao-Usansolo, Galdakao, Bizkaia Spain; 5 Health Services Research on Chronic Patients Network (REDISSEC), Galdakao, Bizkaia Spain

**Keywords:** Pulmonary disease, chronic obstructive, Physical activity, Hospitalizations, Quality of life

## Abstract

**Background:**

Health-related quality of life (HRQoL) should be seen as a tool that provides an overall view of the general clinical condition of a COPD patient. The aims of this study were to identify variables associated with HRQoL and whether they continue to have an influence in the medium term, during follow-up.

**Methods:**

Overall, 543 patients with COPD were included in this prospective observational longitudinal study. At all four visits during a 5-year follow-up, the patients completed the Saint George’s Respiratory Questionnaire (SGRQ), pulmonary function tests, the 6-min walk test (6MWT), and a physical activity (PA) questionnaire, among others measurements. Data on hospitalization for COPD exacerbations and comorbidities were retrieved from the personal electronic clinical record of each patient at every visit.

**Results:**

The best fit to the data of the cohort was obtained with a beta-binomial distribution. The following variables were related over time to SGRQ components: age, inhaled medication, smoking habit, forced expiratory volume in one second, handgrip strength, 6MWT distance, body mass index, residual volume, diffusing capacity of the lung for carbon monoxide, PA (depending on level, 13 to 35% better HRQoL, in activity and impacts components), and hospitalizations (5 to 45% poorer HRQoL, depending on the component).

**Conclusions:**

Among COPD patients, HRQoL was associated with the same variables throughout the study period (5-year follow-up), and the variables with the strongest influence were PA and hospitalizations.

## Background

Chronic diseases are already having and will continue to have not only profound economic, social and individual consequences but also a major impact on the use of health resources and design of new care processes. In this scenario, the patient should play a central role [1].

COPD is a paradigmatic chronic condition. Nowadays, it is understood as a complex heterogeneous multisystem disease, with varying levels of progression and activity in different patients [2, 3]. Because of that, it is necessary to devise tools which evaluate as broadly and comprehensively as possible, the full complexity of the disease.

Health-related quality of life (HRQoL) is commonly used as a predictor of other outcomes such as mortality [4, 5] in COPD patients. Nonetheless, HRQoL is considered an important outcome in itself in many diseases and this should also be the case in COPD. In fact, HRQoL is very often assessed in clinical trials but its use in routine clinical practice and the impact of this use on practice has not been thoroughly studied. Considering that HRQoL gives an overall view of the general clinical condition of a patient, it should be used more frequently in daily clinical practice in COPD. Further, several variables have been related to HRQoL in COPD in various different studies, but these have usually been limited to cross-sectional analysis of a small number of variables.

The aims of this study were to identify variables associated with HRQoL, in a real-life scenario, without any kind of established intervention, and whether these variables initially associated with HRQoL continue to have an influence in the medium-term during follow-up.

## Methods

### Participants and data collection

Patients were recruited after being treated for COPD in one of five outpatient respiratory clinics run by the Respiratory Service of Galdakao Hospital. Patients were consecutively included in the study if they had been diagnosed with COPD for at least 6 months and had been stable for 6 weeks. Other inclusion criteria were forced expiratory volume in one second (FEV1) post-bronchodilator < 80% of the predicted value and a FEV1/forced vital capacity ratio < 70%.

Patients were not eligible for the study if they had been diagnosed with asthma, any other major respiratory disease or cancer, or psychiatric or neurological problems that might hinder effective collaboration. The protocol was approved by the Ethics and Research Committees of the hospital (030906005). All candidate patients were given detailed information about the study and all those included provided written informed consent.

### Study protocol

Sociodemographic variables and smoking habits were recorded. The level of dyspnoea was established using the modified Medical Research Council (mMRC) dyspnoea scale [6]. Comorbidities were identified by reviewing the patients’ entire electronic medical record and summarized using the Charlson comorbidity index [7]. HRQoL was assessed using the validated Spanish version of the Saint George’s Respiratory Questionnaire (SGRQ) [8, 9].

Complete pulmonary function tests included forced spirometry, bronchodilator testing, and body plethysmography, as well as measurements of diffusing capacity of the lung for carbon monoxide (adjusted for hemoglobin) and respiratory muscle strength. These tests were performed in accordance with the standards of the Spanish Society of Respiratory Medicine and Thoracic Surgery (SEPAR) [10]. For theoretical values, we considered those of the European Community for Steel and Coal [11].

Physical activity (PA) was measured using a validated questionnaire [12, 13]. Two 6-min walk tests (6MWTs) were performed according to American Thoracic Society guidelines [14]. Peripheral muscle strength was evaluated in terms of handgrip strength [15].

### Follow-up

Patients were followed up for 5 years. At baseline, interviews were conducted and the aforementioned measurements were performed in all patients; the interview and assessments were then repeated in the first, second, and fifth year of the study period in survivors. No interventions were performed related to this study, and the research team did not take part in patients’ routine care or the treatment of any exacerbations.

Patient medical records and the hospital database on hospitalizations were reviewed at each assessment during this 5-year follow-up period. Vital status was established by reviewing medical records, the hospital database and public death registries. Deaths were considered confirmed if the name, sex, and date of birth on the record matched those of the participant.

### Statistical analysis

The study sample was described using means and standard deviations (SD) for continuous variables and frequencies and percentages for categorical variables.

The beta-binomial (BB) distribution was fitted to the HRQoL scores [16, 17]. The fitting required transforming the original scores to a binomial form as described thoroughly in the literature [18]. The transformation was performed based on a minimum clinically important difference. Jones showed that the estimate for the SGRQ threshold is consistently around 4 units, regardless of the method of estimation and the number of the individuals contributing to the estimate [19]. We developed a transformation process based on the idea that a 4-point change in the SGRQ scores could be considered clinically significant, and hence, we divided the 0 to 100 scale into 4-point length subintervals linking the value of each score to the value of its subinterval [19, 20]. As a result of the transformation process, scores were presented as ordinal scores ranging from 0 to 24. The distribution of the SGRQ scores is shown graphically using histograms.

The annual change in SGRQ scores was estimated using a BB mixed-effects model [21]. One independent model was fitted for each SGRQ component. In this framework, the coefficients estimated using the BB regression model, in particular, the mixed-effects model, are interpreted using odd ratios, equivalent to a logistic regression model, as the probability modelled is that of experiencing a clinically significant change in 1 year in the corresponding SGRQ score. All the measurements were included in the model (one to four for each patient). Covariates included in the multivariate modelling process as fixed effects were: time, sex, age, smoking habit, body mass index, FEV1, residual volume (RV), diffusing capacity of the lung for carbon monoxide (DLCO)*,* 6MWT distance, PA, Charlson comorbidity index, handgrip strength, hospitalizations during the previous year and treatment; while patient was included as a random effect. Finally, only significant covariates were retained in the multivariate models. MacFadden’s rho-squared was calculated for each final model and it was interpreted as the percentage of variability in SGRQ scores that was explained by the model [22]. The importance of each covariate in the models was measured using standardized estimates of the beta coefficients in the model and presented graphically along with the 95% confidence intervals.

Factors related to a clinically significant impairment in HRQoL (4 points in the SGRQ components) over a 1-year period were also studied. The annual change in SGRQ scores was calculated from baseline to 1 year and from 1 to 2 years. Generalized linear mixed models with a logistic link function were used to estimate the probability of an at least 4-point increase in SGRQ score, independently for each component. All the aforementioned covariates were entered into the models.

## Results

The cohort included 543 patients, the majority of whom were men (96%) with moderate obstruction. Other characteristics of the cohort including HRQoL at the baseline and during the follow-up are summarised in Table 1.

**Table 1:** Sociodemographic and clinical characteristics of the cohort during the 5-years of follow-up

Variable	Unit/Category	Baseline *n* = 543	1-year *n* = 480 (88%)	2-years *n* = 428 (79%)	5-year *n* = 324 (60%)
SGRQ	Symptoms	44.5 (22.2)	42.5 (22.4)	43.1 (23.5)	44.1 (23.4)
	Activity	48.7 (24.9)	45.9 (25.0)	46.6 (25.0)	47.4 (25.4)
	Impacts	32.0 (20.9)	30.4 (21.1)	30.1 (20.4)	30.4 (20.9)
Sex	M	522 (96.1)	459 (95.6)	408 (95.3)	308 (95.1)
	F	21 (3.9)	21 (4.4)	20 (4.7)	16 (4.9)
Age	Years	68.3 (8.3)	67.6 (8.4)	67.4 (8.3)	66.2 (8.4)
BMI		28.3 (4.4)	28.3 (5.2)	28.1 (4.4)	27.6 (4.8)
Dyspnea (mMRC)	0	69 (12.7)	85 (17.7)	75 (17.5)	57 (17.6)
	1	264 (48.6)	248 (51.7)	188 (43.9)	134 (41.4)
	2	166 (30.6)	127 (26.5)	142 (33.2)	100 (30.9)
	3–4	44 (8.1)	20 (4.3)	23 (5.4)	33 (10.2)
FEV1	ml	1465(441)	1470(501)	1513(470)	1391(451)
FEV1%	Percentage	55.0 (13.3)	55.2 (16.0)	57.2 (14.7)	54.3 (14.8)
	< 30	18 (3.3)	22 (4.6)	13 (3.0)	14 (4.3)
	30–50	167 (30.8)	131 (27.3)	111 (25.9)	118 (36.4)
	≥50	358 (65.9)	327 (68.1)	304 (71.0)	192 (59.3)
RV%	Percentage	159 (46.9)	158 (47.7)	158 (50.4)	161 (48.4)
DLCO%	Percentage	71.5 (23.5)	75.6 (26.6)	76.7 (26.9)	68.1 (21.9)
Smoking habit/status	Pack/year	46.8(27.3)	46.3(26.4)	46.5(26.0)	46.8(25.9)
	Smoker	114 (23.0)	91 (19.0)	79 (18.5)	58 (17.9)
	Former smoker	414 (76.2)	375 (78.1)	338 (79.0)	256 (79.0)
	Non smoker	15 (2.8)	14 (2.9)	11 (2.6)	10 (3.1)
6mWT	Meters	409 (92)	421 (118)	412 (116)	397 (123)
PA	< 2 h/week	48 (8.8)	44 (9.2)	48 (11.2)	62 (19.1)
	2–4 h/week	110 (20.3)	103 (21.5)	94 (22.0)	60 (18.5)
	> 4 h/week^a^	219 (40.3)	203 (42.3)	165 (38.6)	117 (36.1)
	Work/intense	166 (30.6)	130 (27.1)	121 (28.3)	85 (26.2)
CCI	Points	2.41(1.42)	2.46(1.42)	2.47(1.41)	2.55(1.35)
	< 2	172 (31.7)	145 (30.2)	130 (30.4)	87 (26.9)
	2–3	264 (48.6)	233 (48.5)	208 (48.6)	159 (48.8)
	> 3	107 (19.7)	102 (21.3)	90 (21.0)	79 (24.4)
Strength	Handgrip (kg)	34.1 (9.4)	33.7(10.2)	33.0 (9.6)	32.9(10.4)
Previous hospitalizations	0	427 (78.6)	406 (84.6)	377 (88.1)	234 (72.2)
	1	80 (14.7)	51 (10.6)	30 (7.0)	40 (12.4)
	2	16 (3.0)	14 (2.9)	16 (3.7)	23 (7.1)
	3	9 (1.7)	6 (1.3)	4 (0.9)	14 (4.3)
	> 3	11 (2.0)	3 (0.6)	1 (0.2)	13 (4.0)
Treatment ^b^	0–1	73 (13.4)	57 (11.9)	48 (11.2)	36 (11.1)
	2	121 (22.2)	91 (19.0)	87 (20.3)	56 (17.3)
	3	349 (64.3)	330 (68.8)	293 (68.5)	232 (71.6)

Frequencies and percentages by category for categorical variables, and mean and standard deviation for continuous variables are shown

*BMI* body mass index, *CCI* Charlson comorbidity index, *DLCO* carbon monoxide diffusing capacity, *Dyspnea mMRC* Medical research council scale, *FEV1* forced expiratory volume in the first second, *PA* physical activity, *RV* residual volume, *6mWT* 6-min walking test

^a^ Gardening subjects were included in this category

^b^ Treatment has been classified as follows: 0–1 out of LABA/LAMA; 2 out of LABA/LAMA/ICS; 3 of them LABA+LAMA+ICS

**Fig. 1:**
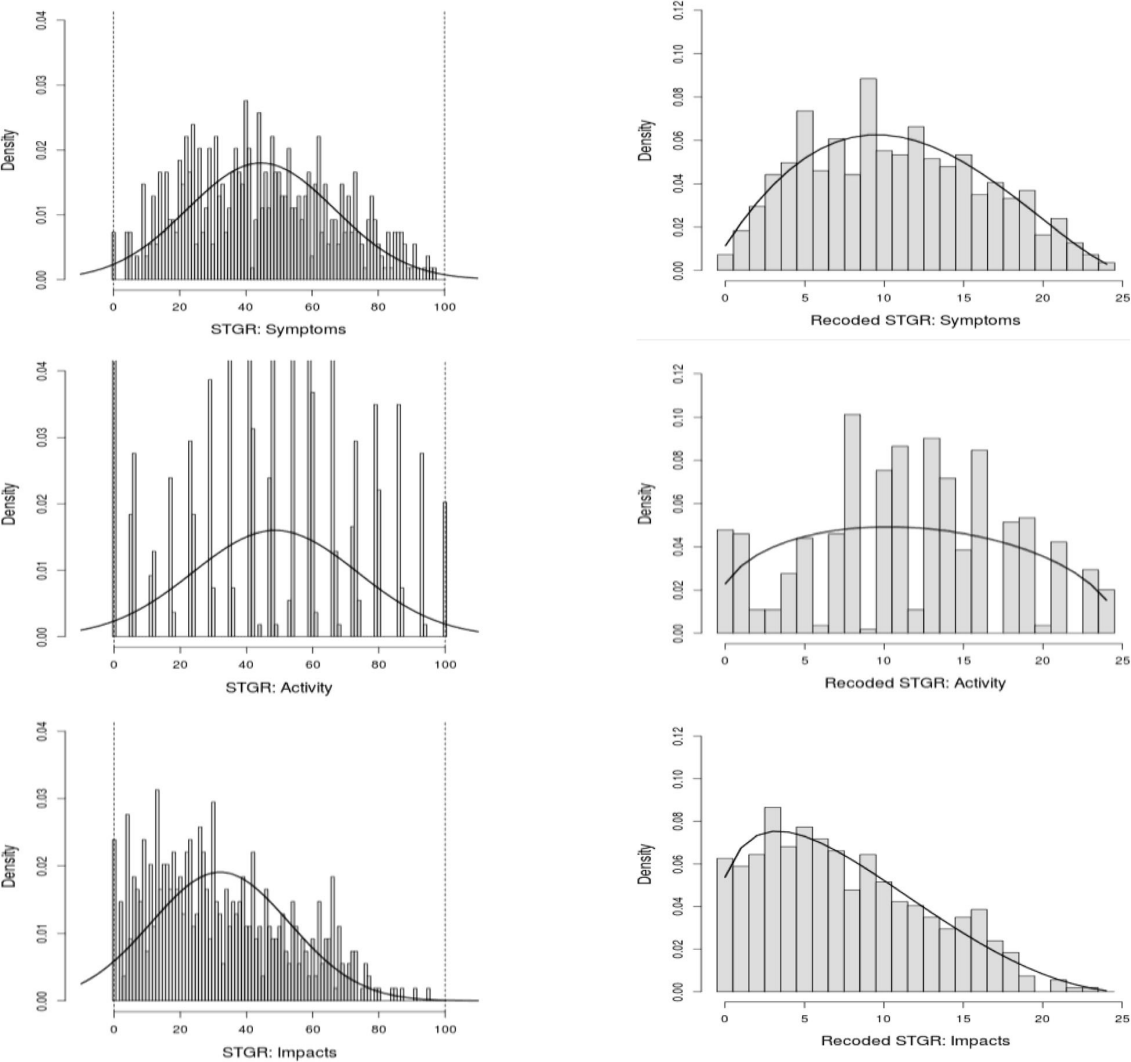
Distribution of the three components of the SGRQ questionnaire at baseline: In the original scale [0, 100] and their fit to the normal distribution (left); and recoded to an ordinal scale from 0 to 25 and their fit to the beta-binomial distribution (right)

Figure 1 shows the distribution of the HRQoL scores at baseline for the three components of the SGRQ. The left side of the Fig. (a) shows the original scores observed and their fit to the normal distribution, and the right side of the Fig. (b) the transformed scores and their fit to the BB distribution. The fit of the BB distribution to the transformed scores was better than that of the normal distribution to the original scores for all the components, especially at the two extremes of the scale.

**Fig. 2:**
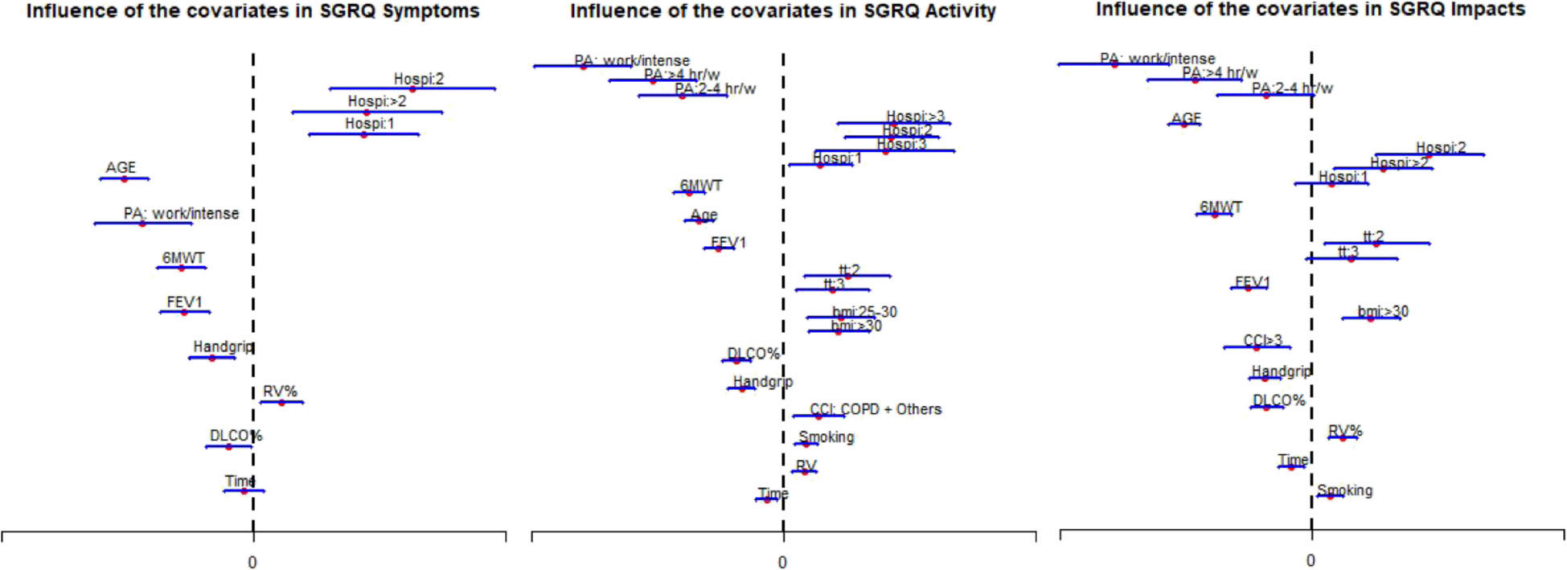
Influence of covariates regarding to the multiple beta-binomial mixed-effects model in the three components of the SGRQ. Estimates and 95% confidence intervals are shown for the beta coefficients. Continuous covariates were standardized in order to allow for comparisons. Categorical variables were included as dummy indicators. Estimates of the reference categories are not displayed (they were zero). Hospi: previous hospitalizations; BMI: body mass index; CCI: Charlson comorbidity index; DLCO: carbon monoxide diffusing capacity; FEV1: forced expiratory volume in the first second; PA: physical activity; RV: residual volume; 6mWT: 6-min walking test; PA: physical activity. BMI: body mass index; tt: treatment (2 out of LABA/LAMA/ICS; 3 of them LABA+LAMA+ICS) CCI: Charlson comorbidity index

Results of the multivariate BB mixed-effects model, adjusted for covariates, are presented in Tables 2, 3, and 4 and Fig. 2 for each of the SGRQ components. The association found remained stable from the measurements taken at the beginning of the study to those taken during the 5-year follow-up in COPD patients that remained alive. PA and hospitalization for COPD exacerbation were the variables most strongly associated with HRQoL in all three components of the SGRQ. Specifically, PA was related to a 13 to 35% better activity and impacts scores of HRQoL, depending on the level of PA, whereas hospitalizations were related to 5 to 45% poorer HRQoL scores, across all three components, depending on the number of hospitalizations.

**Table 2:** Result of the multiple beta-binomial mixed-effects model adjusted by time-dependent covariates for the Symptoms component of the SGRQ questionnaire over time

SGRQ			β	SD(β)	*P*	OR	95%CI
Symptoms	Intercept		2.79	0.269	< 0.001	**–**	–
	Time	(year)	− 0.005	0.010	0.341	**0.991**	(0.971–1.010)
	Age	(5 years)	−0.138	0.013	< 0.001	**0.871**	(0.850–0.893)
	FEV1	(100 ml)	−0.027	0.005	< 0.001	**0.973**	(0.964–0.983)
	RV%	(9%)	0.010	0.004	0.008	**1.010**	(1.003–1.017)
	DLCO%	(11%)	−0.020	0.009	0.028	**0.981**	(0.964–0.998)
	Handgrip	(5 kg)	−0.041	0.011	0.001	**0.960**	(0.939–0.981)
	6mWT	(25 m)	− 0.032	0.005	< 0.001	**0.968**	(0.958–0.978)
	PA	None/Any	–	–	–	–	–
		Work/intense	−0.198	0.044	< 0.001	**0.820**	(0.753–0.893)
	Previous hospitalizations	0	–	–	–	**–**	–
		1	0.197	0.050	< 0.001	**1.217**	(1.105–1.342)
		2	0.284	0.074	< 0.001	**1.328**	(1.148–1.537)
		> 2	0.203	0.068	0.003	**1.224**	(1.073–1.398)
	Sigma^a^		0.742	0.024	–	**–**	–

*DLCO* carbon monoxide diffusing capacity, *FEV1* forced expiratory volume in the first second, *PA* physical activity, *RV* residual volume, *6mWT* 6-min walking test, *SD* standard deviation, *OR* odds ratio, *CI* Confidence interval

^a^ Standard deviation of the random intercept

**Table 3:** Result of the multiple beta-binomial mixed-effects model adjusted by time-dependent covariates for the Activity component of the SGRQ questionnaire over time

SGRQ			β	SD(β)	*P*	OR	95%CI
Activity	Intercept		3.998	0.248	< 0.001	–	–
	Time	(years)	−0.027	0.009	0.002	**0.973**	(0.956–0.990)
	Age	(5 years)	−0.140	0.012	< 0.001	**0.869**	(0.850–0.889)
	FEV1	(100 ml)	−0.040	0.005	< 0.001	**0.961**	(0.952–0.970)
	RV%	(9%)	0.011	0.003	< 0.001	**1.011**	(1.004–1.017)
	DLCO%	(11%)	−0.057	0.009	< 0.001	**0.944**	(0.929–0.961)
	Handgrip	(5 kg)	−0.065	0.010	< 0.001	**0.937**	(0.919–0.955)
	6mWT	(25 m)	− 0.065	0.005	< 0.001	**0.937**	(0.927–0.947)
	Smoking	(10 pack/y)	0.024	0.006	< 0.001	**1.024**	(1.013–1.036)
	BMI	< 25	–	–	–	–	–
		25–30	0.153	0.042	< 0.001	**1.165**	(1.073–1.265)
		> 30	0.158	0.047	< 0.001	**1.171**	(1.068–1.285)
	PA	< 2 h/week	–	–	–	–	–
		2–4 h/week	−0.280	0.062	< 0.001	**0.755**	(0.669–0.854)
		> 4 h/week^a^	−0.363	0.061	< 0.001	**0.696**	(0.618–0.784)
		Work/intense	−0.558	0.068	< 0.001	**0.572**	(0.501–0.654)
	CCI	0–1	–	–	–	–	–
		> 1	0.096	0.036	0.007	**1.101**	(1.026–1.180)
	Treatment^b^	0–1 out of LABA/LAMA	–	–	–	–	–
		2 out of LABA/LAMA/ICS	0.177	0.059	0.003	**1.193**	(1.062–1.340)
		LABA+LAMA+ICS	0.136	0.051	0.008	**1.146**	(1.036–1.267)
	Previous hospitalizations	0	–	–	–	–	–
		1	0.102	0.043	0.021	**1.108**	(1.016–1.208)
		2	0.299	0.066	< 0.001	**1.348**	(1.184–1.535)
		3	0.281	0.098	0.004	**1.325**	(1.094–1.604)
		> 3	0.307	0.079	< 0.001	**1.360**	(1.165–1.587)
	Sigma^c^		0.945	0.030	–	–	–

*BMI* body mass index, *CCI* Charlson comorbidity index, *DLCO* carbon monoxide diffusing capacity, *FEV1* forced expiratory volume in the first second, *PA* physical activity, *RV* residual volume, *6mWT* 6-min walking test, *SD* standard deviation, *OR* odds ratio, *CI* Confidence interval

^a^ Gardening subjects were included in this category

^b^ Treatment has been classified as follows: 0–1 out of LABA/LAMA; 2 out of LABA/LAMA/ICS; 3 of them LABA+LAMA+ICS

^c^ Standard deviation of the random intercept

**Table 4:** Result of the multiple beta-binomial mixed-effects model adjusted by time-dependent covariates for the Impacts component of the SGRQ questionnaire over time

SGRQ			β	SD(β)	*P*	OR	95%CI
Impacts	Intercept		3.150	0.229	< 0.001	**–**	–
	Time	(year)	−0.026	0.008	0.002	**0.974**	(0.959–0.991)
	Age	(5 years)	− 0.167	0.010	< 0.001	**0.846**	(0.829–0.864)
	FEV1	(100 ml)	−0.031	0.004	< 0.001	**0.970**	(0.962–0.978)
	RV%	(9%)	0.013	0.003	< 0.001	**1.013**	(1.007–1.019)
	DLCO%	(11%)	−0.043	0.008	< 0.001	**0.958**	(0.943–0.973)
	Handgrip	(5 kg)	−0.057	0.009	< 0.001	**0.945**	(0.928–0.962)
	6mWT	(25 m)	− 0.053	0.005	< 0.001	**0.949**	(0.940–0.958)
	Smoking	(10 pack/y)	0.015	0.006	0.006	**1.015**	(1.004–1.026)
	BMI	≤ 30	–	–	–	**–**	–
		> 30	0.129	0.032	< 0.001	**1.138**	(1.068–1.212)
	PA	< 2 h/week	–	–	–	–	–
		2–4 h/week	−0.101	0.053	0.056	**0.904**	(0.814–1.002)
		> 4 h/week^a^	−0.256	0.052	< 0.001	**0.774**	(0.700–0.857)
		Work/intense	−0.433	0.061	< 0.001	**0.649**	(0.576–0.731)
	CCI	0–3	–	–	–	**–**	–
		> 3	−0.120	0.037	0.001	**0.887**	(0.825–0.953)
	Treatment^b^	0–1 out of LABA/LAMA	–	–	–	**–**	–
		2 out of LABA/LAMA/ICS	0.141	0.058	0.014	**1.152**	(1.028–1.290)
		LABA+LAMA+ICS	0.087	0.050	0.084	**1.090**	(0.988–1.203)
	Previous hospitalizations	0	–	–	–	–	–
		1	0.043	0.041	0.294	**1.044**	(0.964–1.131)
		2	0.257	0.060	< 0.001	**1.293**	(1.149–1.454)
		> 2	0.156	0.054	0.004	**1.169**	(1.050–1.300)
	Sigma^c^		0.951	0.030	–	–	–

*BMI* body mass index, *CCI* Charlson comorbidity index, *DLCO* carbon monoxide diffusing capacity, *FEV1* forced expiratory volume in the first second, *PA* physical activity, *RV* residual volume, *6mWT* 6-min walking test, *SD* standard deviation, *OR* odds ratio, *CI* Confidence interval

^a^ Gardening subjects were included in this category

^b^ Treatment has been classified as follows: 0–1 out of LABA/LAMA; 2 out of LABA/LAMA/ICS; 3 of them LABA+LAMA+ICS

^c^ Standard deviation of the random intercept

Pulmonary function was associated with all HRQoL components, with an approximately 5% improvement in HRQoL score for each 100 ml increase in FEV1 and 11% increase in DLCO. These results were of a similar magnitude to the improvement seen with each 5-kg increase in handgrip strength and 25-m increase in the 6MWT. A bigger deterioration of HRQoL was caused by every 9% increase in RV, but its level of influence was lower than that of FEV1 or DLCO.

Age showed a positive relationship with HRQoL in all the components of the SGRQ.

The amount of random variance in the SGRQ scores explained by the model ranged from 24% for symptoms to 44% for activity and impacts. The probability of clinically significant impairment in 1 year was estimated to be 0.33 for the impacts component and 0.36 for the activity component, and it did not depend on any of the covariates. On the other hand, the probability of clinically significant impairment in 1 year for the symptoms component depended only on the presence of cardiovascular disease (CVD) and was estimated to be 0.44 for patients without CVD; while the OR for patients with CVD compared to those without CVD was 1.41 (95% CI (1.05–1.90).

## Discussion

1. Important variables in characterization and prognosis in COPD (PA, hospitalizations, exercise capacity, muscular strength, FEV1, DLCO, smoking habit, and age) were associated with HRQoL from the beginning to the end of the 5-year follow-up.
2. The HRQoL was well fit by the BB distribution, justifying the use of BB regression for the analysis instead of traditional normal distribution and linear models usually used in this kind of study.
3. PA and hospitalizations were the factors with the strongest influence on HRQoL.

Many studies have identified various different factors associated with HRQoL in COPD patients; however, what has not been thoroughly studied is whether or not these factors maintain their influence over time. Moreover, these previous cross-sectional studies tend to have only assessed HRQoL at the start of the study and take it for granted that the data were normally distributed. In our study, we carried out four assessments of HRQoL during the 5 years of follow-up, all of which were included in the analysis and the BB distribution was applied to the data as a whole. Previous research found the BB regression approach to perform better for analysing HRQoL data than the standard regression approaches based on the normal distribution of the outcome [16]. Further, in this particular case, we have shown that the empirical distribution of the SGRQ scores (Fig. 1) is much better fitted by the BB than the normal distribution. Moreover, the easy interpretation of the results in terms of OR is an added advantage of the BB regression compared to the classical linear regression approach. Extension of the BB regression model to the BB mixed-effects model, recently proposed in the literature [21], allowed us to use all the repeated measurements over time to estimate the effect of relevant covariates on HRQoL. Considering that not only HRQoL was measured over time, but also that many covariates were time dependent, effective statistical analysis should include all the repeated measurements, considering the longitudinal design. Thanks to the BB mixed-effects approach, 543 patients have been analysed based on 1775 correlated records, providing robustness to the results.

Although the amount of random variance in the SGRQ scores explained by the models seems low (0.24–0.44), it should be noted that values of rho-squared tend to be considerably lower than those of the traditional R-squared in linear regression. In fact, McFadden [23] stated that values of 0.2 to 0.4 for rho-squared represent an excellent fit.

General speaking, PA was the factor with the strongest positive influence on HRQoL in our cohort. Changes in PA measured by questionnaires or by accelerometers have previously been shown to be related to changes in HRQoL [24, 25]. In our study, we found that it was the most important factor related to HRQoL during the whole follow-up and especially for the activity and impacts components of the SGRQ. Previously, our group had shown that there was an association between changes in PA and changes in HRQoL. Our current finding is complementary to those results [24], in that PA appears as a predictor of HRQoL throughout the natural history of the disease, regardless of its duration and severity.

The other variable with a major influence was the occurrence of hospitalizations. In this case, it was the factor with the most strongly growing negative influence in HRQoL from the first admission. An independent association between number of hospitalizations and HRQoL was previously shown by our group [26], in agreement with other authors [27]. In the current study, the relevance of hospitalizations in HRQoL is put into perspective compared with other variables.

Previous research has found a relationship between the number of exacerbations and HRQoL measured by SGRQ [28], but in this study, only 16% were severe exacerbations, and it was the patients with a high frequency of exacerbation (between 3 and 8/year) who had a clinically and statistically significantly poorer HRQoL [28]. Further, the current study adds weight to the known crucial importance of severe exacerbation in HRQoL [29], and the resulting need to make prevention of severe exacerbation a priority target in COPD patients. On the other hand, the relationship between PA and hospitalization is well known [12, 30, 31] and our study helps to close the loop on the relationship between PA, hospitalizations and HRQoL.

It has been known for several years that FEV1 is related to HRQoL, especially as severity increases, FEV1 50% being indicated as a turning point associated with marked impairment of HRQoL [32]. In our study DLCO had a positive relationship with all the domains of SGRQ, but this influence was lower than that of FEV1%, as has been published [33]. On the other hand, other variables such as the body mass index, obstruction, dyspnoea and exercise index (BODE index) showed better correlation with HRQoL (SGRQ) than FEV1 alone [34]. In our study, some of the variables included in the BODE index were also related to HRQoL, but they did not have as strong an influence as that of PA or hospitalizations. It is worth mentioning that muscular strength (handgrip) had a positive influence on all the components of the SGRQ, as has been pointed out previously [35].

We want to emphasize that all the variables found to be associated with HRQoL have remained the same throughout the follow-up years. Ferrari et al. studied 95 COPD patients that remained alive in a 3-year follow-up study and found that the variables related to HRQoL were BODE index and, in particular, dyspnoea at the start of the study, but BODE index and age 3 years later. When the components of the BODE index were considered, the predictors of HRQoL were dyspnoea at baseline, and dyspnoea, FEV1 and exacerbations (moderate or severe) at 3 years [36]. This contrasts with our observation that the variables involved as predictors of HRQoL did not change throughout the 5-year follow-up. The small number of patients they included in their study may have influenced their results.

In our study, age was related to HRQoL. In fact, older people had better HRQoL after controlling for several other variables. These results are in agreement with those of another cohort [37], and while some authors found opposite results [38], they made no mention of controlling for confounders. The reasons why HRQoL improves as age increases have not been clearly identified. In our opinion, it is probably a matter of people’s expectations changing as they get older and/or they progressively get used to living with the disease.

It might be expected that cardiovascular comorbidities would play an important role in HRQoL of COPD patients [39], but this does not seem to be observed, unlike for other comorbidities, such as depression, which have been shown to contribute strongly to HRQoL [40]. In a European cross-sectional study, a cut-off of three comorbidities established significant differences in HRQoL, and though COPD patients with cardiovascular comorbidities had worse scores in HRQoL (SGRQ), the minimal clinically important difference was not reached [41]. In our study, comorbidities seem not to have influenced the HRQoL of the cohort. This could be explained by the SGRQ, a specific questionnaire, not being able to capture the whole impact of comorbidities in these patients. It is plausible that generic questionnaires are the best instruments to capture the influence of comorbidities on the HRQoL of COPD patients [42, 43]. For this reason, some authors have suggested that a combination of generic and specific questionnaires best reflect the impact of comorbidities [42]. This is an issue that should be considered in daily clinical practice.

Since the study was an observational study, we did not establish whether the inhaled treatment of patients matched that recommended in guidelines, and hence, our results reflect that using more than two inhaled medications was associated with a better quality of life. Further, a limitation of the study is that our findings are obtained from a predominantly male cohort and hence might not fully apply to women. We included several variables with a recognized impact on COPD patients, but there are bound to be other variables that also have an influence. On the other hand, having explored how the SGRQ works and what we can expect from this kind of instrument, it is clear that use of this questionnaire is not feasible in clinical practice, and hence, there is a need to assess the properties over time of other questionnaires used in clinical practice (e.g., the COPD Assessment Test, and the Clinical COPD Questionnaire) to confirm whether their behaviour is similar to that demonstrated for SGRQ in this study. Lastly, survival bias could have minimized the influence of the variables on the final results of the study. Strengths of the study include that we have studied a range of variables and how they maintain their influence on HRQoL, regardless of disease duration.

## Conclusions

HRQoL summarized the general clinical condition of COPD patients, mirroring the influence of many important related variables and the same set of variables continued to have an influence over time. Two key modifiable factors that influenced HRQoL were PA and hospitalizations for exacerbation. Further, it is worth investigating a different methodological approach to HRQoL based on the BB.

## Data Availability

The datasets used and/or analysed during the current study are available from the corresponding author on reasonable request.

## Abbreviations

BB: Beta-binomial distribution
BODE index: (body mass index, Obstruction, Dyspnea, Exercise index)
CVD: Cardiovascular disease
Dyspnea mMRC: Modified Medical Research Council scale
FEV1: Forced expiratory volume in one second
HRQoL: Health-related quality of life
PA: Physical activity
SGRQ: Saint George’s Respiratory Questionnaire
6MWT: 6-min walk test
